# A 5 year retrospective analysis of common intestinal parasites at Poly Health Center, Gondar, Northwest Ethiopia

**DOI:** 10.1186/s13104-019-4735-9

**Published:** 2019-10-25

**Authors:** Meseret Ayelgn, Ligabaw Worku, Getachew Ferede, Yitayih Wondimeneh

**Affiliations:** 1 University of Gondar Comprehensive Specialized Hospital Laboratory, University of Gondar, Gondar, Ethiopia; 2 Department of Medical Parasitology, School of Biomedical and Laboratory Sciences, College of Medicine and Health Sciences, University of Gondar, Gondar, Ethiopia; 3 Department of Medical Microbiology, School of Biomedical and Laboratory Sciences, College of Medicine and Health Sciences, University of Gondar, Gondar, Ethiopia

**Keywords:** Intestinal parasite, Trend prevalence, Gondar, Ethiopia

## Abstract

**Objective:**

Intestinal parasites are present throughout the world in varying degrees of prevalence due to many factors. The aim of this study was to determine the 5-year trend prevalence of intestinal prevalence among patients who had been suspected for intestinal parasite infections. A retrospective study was conducted from 2009 to 2013 at Poly Health Center Gondar, Northwest Ethiopia. Samples were examined using direct saline wet mount methods. Statistical analysis was done using SPSS version 20 software and a P-value of < 0.05 was considered statistically significant. The results were presented in tables and graphs.

**Results:**

During the study period, a total of 13,329 stool samples were requested for intestinal parasite diagnose and 5510 (41.3%) laboratory-confirmed cases were reported with a fluctuating trend. Ten different parasites were reported in each year with *Entamoeba histolytica/dispar* (16.8%) being the predominant parasite followed by *Giardia lamblia* (11.4%) and *Ascaris lumbricoides* (6.7%). Both males (49%) and females (51%) were equally affected (P = 0.14). The intestinal parasite was reported in all age groups in the area but the highest and the lowest prevalence were reported in age groups of 20–29 years and 40–49 years, respectively (26.5% vs 6.4%) (P < 0.001).

## Introduction

Intestinal parasites are present throughout the world in varying degrees of prevalence and tend to be most prevalent in developing countries [1]. This is due to ecological and socioeconomic factors and differences in human behaviors and sanitation particularly in the tropics and subtropics, crowded population and deficient sanitation as well as lack of potable water [2]. According to the World Health Organization (WHO) report, there are about 800–1000 million cases of Ascariasis, 200 million cases of Giardiasis and 500 million cases of Amoebiasis globally [3–5]. Similarly, in sub-Saharan African countries, 200–500 million people are affected with at least one or more species of these intestinal parasites [6]. The intestinal parasitic infection affects the health status of an individual’s mainly physical and mental development. It leads to malnutrition, anemia, stunting, and cognitive impairment, thus remains to be a major public health problem globally [7, 8].

Among African countries, intestinal parasites have been widely distributed in Ethiopia due to poverty, lack of environmental sanitation and ignorance of simple health promotion practice. According to the Ethiopian Ministry of Health, the prevalence rate of infection with intestinal parasite is as high as 70% and there is also a high rate of multiple infections reported from many urban and rural areas [9–19]. But in the study area, there is no such type of recent data that shows the magnitude of the problem. Therefore, the aim of this study was to determine the trend prevalence of intestinal parasites among patients who were suspected for intestinal parasite infections and gave stool samples for laboratory diagnosis.

## Main text

### Materials and methods

#### Study design, period and area

A retrospective study was conducted from 2009 to 2013 at Poly Health Center in Gondar town, Northwest Ethiopia. Gondar town is located 747 km (kms) north of Addis Ababa, the capital city of the country. The town has a latitude and longitude of 12°36′N 37°28′E with an elevation of 2133 meters above sea level. Gondar has mid-altitude climate and an average annual maximum and minimum temperatures of 27 °C and 16 °C, respectively. A total population of the town is about 207, 044, of whom 98,120 were men and 108,924 were female and the population of the district is predominately Amhara, Orthodox Christianity being the main religion. The town administration has twenty-three urban and eleven rural kebeles [20].

#### Study design

A retrospective study was conducted to determine the 5-year trend prevalence of intestinal parasite by reviewing the laboratory registration book that contains wet mount reports for the detection of intestinal parasite at Poly Health Center.

#### Study participants and data collection

The study participants were all individuals who were suspected for intestinal parasite infections and gave a stool sample for laboratory diagnosis during the study period. Socio-demographic and laboratory results were reviewed from the laboratory registration book and collected by worksheet designed for this purpose. Proper stool samples were collected with a labeled clean, dry, leak-proof and sterile plastic container. In the study area, the only method to diagnose intestinal parasite infections was saline wet technique. According to standard operating procedure of the health center, direct stool examinations were done using saline wet mount technique within 30 min of sample collection. The collected stool samples mostly diagnosed for parasites like *Entamoeba histolytica/dispare* (*E. histolytica/dispare*), *Giardia lamblia* (*G. lamblia*), *Ascaris lumbricoides* (*A. lumbricoides*), Hookworm, *Trichuris trichiura* (*T. trichiura*), *Hymenolepis nana* (*H. nana*), *Enterobius vermicularis* (*E. vermicularis*), *Taenia* species, Sc*histosoma mansoni* (*S. mansoni*), *Strongyloid stercularis* (*S. stercularis*) and other intestinal parasites. After stool examinations completed, infected patients were treated based on the national guide line.

#### Data processing and analysis

Data collected from the laboratory registration book using the worksheet were first cleaned manually and entered and analyzed by using SPSS version 20 software. A Chi-square test was employed to compare the proportion of intestinal parasite isolates with patients’ demographic information. P-value < 0.05 was considered statistically significant.

### Results

#### Socio-demographic characteristics and its relation with intestinal parasites infection at Poly Health Center

During the study period (2009–2013), a total of 13,329 stool samples were requested for intestinal parasite diagnosis in the study area. Of the total patients requested, 6427 (48.2%) were males while 6902 (51.8%) were females. The age of the study participants ranges from 1 to 81 years. During the study period, the overall prevalence of the intestinal parasites was 5510/13,329 (41.3%). Of the total positive samples in the last 5 years in the health center, there is no statistically significant difference in the parasitic infection rates among males and females [2699/5510 (49%) vs 2811/5510 (51%) (P = 0.14), respectively]. Intestinal parasites were also reported in all age groups in the area but there is statistically significant difference in the proportion of parasitic infections in different age groups. The highest intestinal parasitic infection was reported in the age group of 20–29 years but the least infection rate was reported in the age group of 40–49 years (26.5% vs 6.4%) (P < 0.001).

During the study period, ten different species of parasites were identified. Of these, *E. histolytica/dispar* being the predominant parasite followed by *G. lamblia* and *A. lumbricoides* with a prevalence of 40.7%, 27.5% and 16.2%, respectively. The other identified parasites were *H. nana*, *E. vermicularis*, *Taenia* species, *S*. *mansoni*, *S. stercularis*, *T. trichiura* and Hookworm (Table 1).

**Table 1:** Distribution of different intestinal parasite species in relation to sex and age groups at Poly Health Center, Gondar, Northwest Ethiopia, 2009–2013

Type of parasite	Sex No (%)			Age groups No (%)						
	Male	Female	Total	≤ 9	10–19	20–29	30–39	40–49	≥ 50	Total
*A. lumbricoides*	422 (47.4)	*469* (*52.6*)	891 (16.2)	202 (22.7)	*240 (26.9)*	228 (25.6)	102 (11.4)	60 (6.7)	59 (6.6)	891 (16.2)
Hookworm	*102* (*55.7*)	81 (44.3)	183 (3.3)	*45 (24.6)*	34 (18.6)	55 (30.1)	23 (12.6)	11 (6.0)	15 (8.2)	183 (3.3)
*T. trichiura*	21 (44.7)	*26 (55.3)*	47 (0.9)	*16 (34.0)*	11 (23.4)	9 (19.1)	5 (10.6)	2 (4.3)	4 (8.5)	47 (0.9)
*S. mansoni*	*81* (*60.9*)	52 (39.1)	133 (2.4)	26 (19.5)	*48 (36.1)*	39 (29.3)	12 (9.1)	3 (2.3)	5 (3.8)	133 (2.4)
*S. stercularis*	47 (45.6)	*56 (54.4)*	103 (1.9)	15 (14.7)	28 (27.2)	*33 (32.0)*	14 (13.6)	6 (5.8)	7 (6.8)	103 (1.9)
*H. nana*	113 (49.6)	*115 (50.4)*	228 (4.1)	*105 (46.1)*	70 (30.7)	35 (15.4)	11 (4.8)	3 (1.3)	4 (1.8)	228 (4.1)
*Taenia Species*	13 (40.6)	*19 (59.4)*	32 (0.7)	5 (15.6)	*9 (28.1)*	7 (21.9)	4 (12.5)	2 (6.3)	5 (15.6)	32 (0.7)
*E. vermicularis*	66(49.6)	*67 (50.4)*	133 (2.4)	*41 (30.8)*	33 (24.8)	27 (20.3)	15 (11.3)	6 (4.5)	11 (8.3)	133 (2.4)
*G. lamblia*	746 (49.2)	*770 (50.8)*	1516 (27.5)	*460 (30.3)*	275 (18.1)	381 (25.1)	179 (11.8)	105 (6.9)	116 (7.7)	1516 (27.5)
*E. histolytica/dispar*	1088 (48.5)	*1156 (51.5)*	2244 (40.7)	457 (20.4)	498 (22.2)	*645 (28.4)*	276 (12.3)	154 (6.9)	214 (9.5)	*2244 (40.7)*
Over all total	2699 (49)	*2811 (51)*	5510 (100)	1372 (24.9)	1246 (22.6)	*1459 (26.5)*	641 (11.6)	352 (6.4)	440 (8.0)	5510 (100)

The highest frequency of the intestinal parasites infection in relation to sex and age groups is showed in italics

As to the distribution of different intestinal parasite species in relation to the sex of the study participants, except for Hookworm and *S. mansoni species,* females were more affected than males. In relation the age groups, the *F. histolytica/dispar*, Hookworm and *S. stercularis*, were higher in the 20–29 age group with the prevalence rate of 645/2244 (28.4%), 55/183 (30.1%) and 33/103(32.0%), respectively. The age group < 9 was more affected by *G. lamblia*, *H. nana*, *E. vermicularis* and *T. trichiura* with the prevalence of 460/1516 (30.3%), 105/228 (46.1%), 41/133 (30.8%) and 16/47 (34.0%), respectively. and 10–19 age group where affected by *A. lumbricoide*, *S. mansoni* and *Taenia* species (Table 1).

#### Annual trends of intestinal parasite prevalence at Poly Health Center

**Fig. 1:**
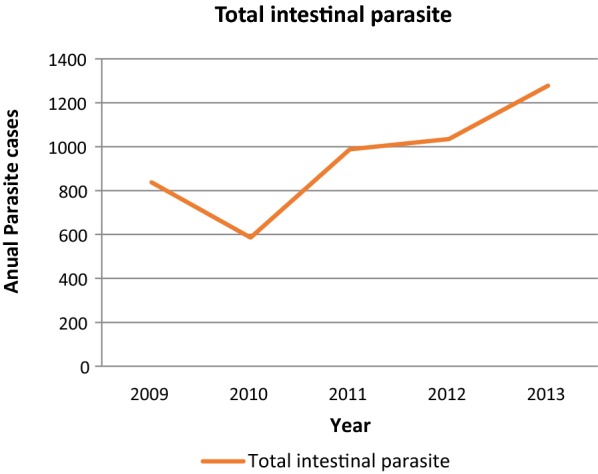
Annual trend of total intestinal parasite cases at Poly Health Center, Gondar, Northwest Ethiopia, 2009–2013

There was a slight fluctuating trend of intestinal parasites within the last 5 years with the maximum number laboratory confirmed cases of intestinal parasite were reported in 2009 with the prevalence of 838/1583 (52.9%) and the minimum, 586/1973(29.7%), were reported in 2010 (Fig. 1).

**Fig. 2:**
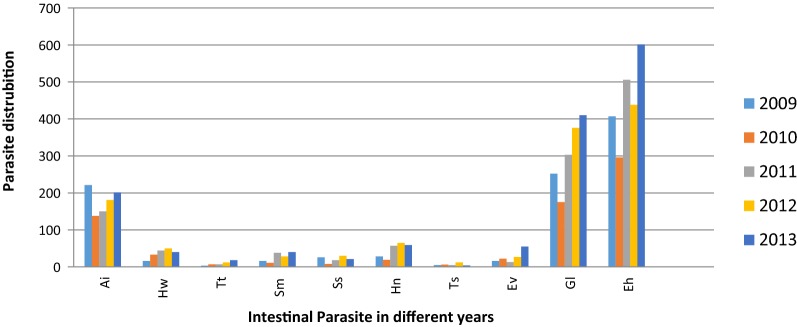
Intestinal parasites distribution at Poly Health Center, Gondar, Northwest Ethiopia, 2009–2013. *Al*, *Ascaris lumbricoide*, HW, Hook worm; *Tt*, *Trichuris trichiura*; *Sm*, *Shistosoma mansoni*; *Ss*, *Strongloide stericularis*; *Hn*, *Hymenolepis nana*; *Ts*, *Taenia* Species; *Ev*, *E.vermicularis*; *Gl*, *Giardia lamblia*; *Eh*, *Entamoeba histolytica/dispar*

As to the prevalence of individual parasitic species in relation to the different years in the study period, *E. histolytica/dispar*, *G. lamblia, S. mansoni*, *S. stercularis* and *T. trichiura* were higher in 2013. *Hymenolepis nana*, *E. vermicularis*, *Taenia* species and Hookworm were higher in 2012 while *A. lumbricoides* was higher in 2009 (Fig. 2).

### Discussion

Monitoring and observing the trend distribution of intestinal parasitic infections in a certain community is a precondition for planning and evaluation of the existing intervention program. In line with this view, the present study attempted to assess 5-year retrospective analysis of common intestinal parasites at Poly Health Center, Gondar, Northwest Ethiopia. The results of the study showed the occurrence of several intestinal parasites of public health importance among the community.

Although the present study used only wet mount technique that might have less sensitivity to diagnose light intestinal parasitic infections, the overall prevalence of intestinal parasite was 41.3% which was higher than other studies conducted in different part of Ethiopia; Bale-Robe (6.23%), Mojo Health Center (9.3%), Wonago Health center (16.6%) [21–23] and other countries’ report [24–35]. These differences in prevalence could be due to the use of different diagnostic methods and socio-demographic differences of the study subjects or it might be due to a reflection of the local endemicity and geographic condition of the study area.

*Entamoeba histolytica/dispar* (40.7%) was the most prevalent intestinal parasite but it was lower than the previous study in Wonago Health center (53.8%) in Ethiopia [23]. However, the prevalence in the present study was higher than that of the studies done in Bale-Robe (26.3%) [21] and other countries, Palestine (16.3%) [24], Palajunoj Valley of Guatemala (16.1%) [25], Osmangazi University Medical Faculty in Turkey (31%) [29] and Kenitra in Morocco (23.7%) [35]. The prevalence of this study was also significantly higher than the studies conducted in Mojo Health Center (4.0%) in Ethiopia [21], Uludag University Medical School Hospital (5.4%), Istambul (0.05%), Tokat public Laboratory (0.8%), Dukuz Eylul University Medical Faculty Hospital (0.3%), Sivas municipality (3.7%) in Turkey, Riyadh Region (0.14%) in Saudi Arabia and Tehran (1.1%) in Iran [25–28, 30–32]. These differences might be due to the difference in diagnostic methods as discussed earlier. In addition, might be due to the difference in getting safe drinking water.

In the present study, *G. lamblia* (27.5%) was the second most prevalent intestinal parasite and *A. lumbricoides* (16.2%) as the leading helminthic parasite followed by *H. nana*, Hookworm, *E. vermicularis, S. mansoni*, with the prevalence of 4.1%, 3.3%, 2.4%, 2.4%, respectively and the least prevalence *Taenia* species (0.7%) which was differ from the studies done in Ethiopia [21–23] and others countries [24–32, 35]. In addition to the possible factors like difference in diagnostic approach and the sources of safe drinking water discussed earlier, the differences in the proportions of these parasites in different studies might be also due to the difference in socio-economic level, sanitary/hygienic status, occupation, and cultural activities and nutritional factors of the study populations.

The result of our study revealed that a slight fluctuating overall trend of intestinal parasitic infection was observed in the study area. A decreasing number of infections occurred in 2010 compared with reported in 2009. However, there was a gradual increment of a number of cases from 2011 to 2013 with higher intestinal parasite infection being reported in 2013. Similar studies in Ethiopia [22], Palestine [24], Turkey [26] and Saudi Arabia [31] reported a fluctuating trend of overall intestinal parasite infection. However, our study finding differed from the study conducted in Ethiopia [21] and Turkey [25] which was increasing and Turkey [29], Italy [33] and Morocco [34] which was decreasing trend parasite infection. The possible reason might be due to the difference prevention control strategy of different locality in a country and between countries, geographical differences, and the living condition of the study subjects. In the present study area, there is no prevention and control strategy except treating patients suspected and diagnosed for intestinal parasitic infections.

Out of the total data review in the last 5 years in the health center, males and females were equally affected for intestinal parasites infection except for Hookworm and *S. mansoni.* Males were more affected than females by Hookworm and *S. mansoni*. This study is consistent with the study done in Uludag University Medical School Hospital and Istanbul in Turkey [25, 26]. But in other studies in Mojo Health Center and Monago Health Center in Ethiopia [22, 23], Tokat Public laboratory, Osmangazi University Medical Faculty in Turkey [27, 29], females were more affected. The differences in the proportion of parasitic infection between males and females might be due to the difference in their daily activities. In the study area, males are mostly involved in outdoor activities like farming and swimming. These activities might expose more males to the soil-transmitted parasitic infection and intestinal flukes.

Regarding the age groups, 20–29 years were highly affected groups, with a prevalence rate of (26.5%), followed by < 9-year-olds (24.9%). *Entamoeba histolytica/dispar* (28.4%), Hookworm (30.1%), and *S. stercularis* (32.0%) were higher in the earlier age groups. But the *G. lamblia* (30.3%), *H. nana* (46.1%), *E. vermicularis* (30.8%) and *T. trichiura* (34.0%) were higher in the later age groups. The 10–19 age groups were also affected by *A. lumbricoide*, *S. mansoni,* and *Taenia* species. The higher overall distribution of *E. histolytica/dispar*, Hookworm and *S. stercularis in* adolescence groups in the present study was in agreement with other studies in Ethiopia [21, 22] and other countries [25, 26, 28, 34]. But it differed from other studies done in the Wonago health center in Ethiopia and Catholic University Hospital in Italy [23, 33]. This may be probably due to the difference in hygienic status. The highest rate of infection was in the childhood, adolescent and oldest group. These indicate that these groups of persons are highly active and they are less likely to keep their hygiene. They might have close contact with pollutants with helminths infested soil or water bodies to play.

### Conclusions

Intestinal parasites were highly prevalent in the study area and fluctuating trend among the study subject for the past 5 years. *Entamoeba histolytica/dispar* was the predominant parasite. Males were more affected by Hookworm and *S. mansoni*. In the present study, the parasitic infection was mostly common in the age groups of 20–29 years. This indicates the importance of establishing proper infection and prevention strategies by considering different factors like daily activities that exposed to parasitic infections. There should be also a strong surveillance system in the study area to monitor this high prevalence of parasitic infections in the populations.

## Limitation of the study

Due to the nature of the study, we were unable to get risk factor information’s like the quality of water supply, environmental sanitation, hygiene practice at both individual and community levels. Hence, we are unable to correlate with the main parasites obtained in the present study. Since the laboratory method employed for the identification of intestinal parasites in the study area was saline wet mount preparation, we are unable to differentiate *E. histolytica* and *E. dispar* due to their morphological similarities. In addition, using the wet mount method only might not be enough to diagnose light parasitic infections as compared to like concentration methods. Hence, the magnitude of parasitic infection in the study area might be underestimated due to the nature of the study.

## Data Availability

All the relevant data analyzed in this study are presented in the manuscript and there are no additional datasets and materials.

## Abbreviations

WHO: World Health Organization
Kms: kilometers
*E. histolytica/dispare*: *Entamoeba histolytica/dispare*
*G. lamblia*: *Giardia lamblia*
*A. lumbricoides*: *Ascaris lumbricoides*
*T. trichiura*: *Trichuris trichiura*
*H. nana*: *Hymenolepis nana*
*E. vermicularis*: *Enterobius vermicularis*
*S. mansoni*: *Schistosoma mansoni*
*S. stercularis*: *Strongyloide stercularis*
